# Lack of association between clinical and ultrasound measures of disease activity in rheumatoid arthritis remission

**DOI:** 10.1177/1759720X20915322

**Published:** 2020-05-11

**Authors:** Kenneth F. Baker, Ben Thompson, Dennis W. Lendrem, Adam Scadeng, Arthur G. Pratt, John D. Isaacs

**Affiliations:** Musculoskeletal Research Group, Translational and Clinical Research Institute, Newcastle University, Newcastle upon Tyne, UK; Musculoskeletal Unit, Freeman Hospital, Newcastle upon Tyne Hospitals NHS Foundation Trust, Newcastle upon Tyne, UK; Musculoskeletal Research Group, Translational and Clinical Research Institute, Newcastle University, Newcastle upon Tyne, UK; Musculoskeletal Unit, Freeman Hospital, Newcastle upon Tyne Hospitals NHS Foundation Trust, Newcastle upon Tyne, UK; Musculoskeletal Research Group, Translational and Clinical Research Institute, Newcastle University, Newcastle upon Tyne, UK; Musculoskeletal Unit, Freeman Hospital, Newcastle upon Tyne Hospitals NHS Foundation Trust, Newcastle upon Tyne, UK; Musculoskeletal Unit, Freeman Hospital, Newcastle upon Tyne Hospitals NHS Foundation Trust, Newcastle upon Tyne, UK; Musculoskeletal Research Group, Translational and Clinical Research Institute, Newcastle University, Newcastle upon Tyne, UK; Musculoskeletal Unit, Freeman Hospital, Newcastle upon Tyne Hospitals NHS Foundation Trust, Newcastle upon Tyne, UK; Musculoskeletal Research Group, Translational and Clinical Research Institute, Faculty of Medical Sciences, Newcastle University, William Leech Building, Framlington Place, Newcastle upon Tyne, NE2 4HH, UK; Musculoskeletal Unit, Freeman Hospital, Newcastle upon Tyne Hospitals NHS Foundation Trust, Newcastle upon Tyne, UK

**Keywords:** greyscale, power Doppler, remission, rheumatoid arthritis, synovitis, ultrasound

## Abstract

**Objectives:**

The objective of this study was to assess the prevalence of ultrasound (US) abnormalities and association with clinical parameters in rheumatoid arthritis (RA) clinical remission.

**Methods:**

Patients with established RA in clinical remission (DAS28-CRP < 2.4) taking conventional synthetic disease-modifying anti-rheumatic drugs were recruited as part of the Biomarkers of Remission in Rheumatoid Arthritis (BioRRA) Study. In addition, patients from the Newcastle Early Arthritis Clinic (NEAC) with early active RA (DAS28-CRP > 2.4) or seronegative non-inflammatory arthralgia (NIA) were studied as positive and negative controls, respectively. The association between individual dependent variables (synovial power Doppler and greyscale, tenosynovial greyscale, and erosions) and clinical parameters was assessed by multivariate ordinal logistic regression, with adjustment for multiple testing.

**Results:**

A total of 294 patients were included: 66 RA in remission, 146 active RA, and 82 NIA. Within the active RA group, significant associations were observed between swollen joint count and higher total synovial greyscale score (OR 1.17 95% CI 1.08–1.26, *p* < 0.001) and higher total synovial power Doppler score (OR 1.20, 95% CI 1.12–1.30, *p* < 0.001). No significant associations were observed for the NIA group. In the RA remission group, US abnormalities were frequently observed and comparable for both DAS28-CRP and 2011 ACR/EULAR Boolean remission, with no significant association with clinical parameters identified.

**Conclusion:**

We observed widespread subclinical US findings in RA patients in clinical remission, even when remission is defined using the stringent ACR/EULAR Boolean criteria. In contrast to active disease, synovial power Doppler failed to show significant association with any of the clinical parameters in RA remission. Our results suggest that clinical and US examinations are non-overlapping in evaluating RA remission, challenging the proposition of US-driven management strategies in this setting.

## Introduction

Rheumatoid arthritis (RA) is treated with disease-modifying anti-rheumatic drugs (DMARDs) which are escalated until a predefined target of disease activity is achieved, ideally disease remission.^1^ Currently, remission is defined clinically through the use of composite clinical scores such as the disease activity score in 28 joints (DAS28),^2^ and, more recently, the 2011 American College of Rheumatology (ACR)/European League Against Rheumatism (EULAR) RA remission criteria.^3^ Nevertheless, some patients who achieve clinical remission can still accumulate joint erosions,^4^ suggesting a degree of subclinical synovitis that is captured imprecisely by clinical assessment alone.

The past two decades have witnessed an increased use of musculoskeletal ultrasound (US) in the diagnosis and management of RA. Active synovitis, visible as power Doppler (PD) change on US imaging, is predictive of future arthritis flare and joint erosion.^4^ However, the extent to which US parameters are associated with the ‘depth’ of remission – especially fulfilment of ACR/EULAR remission criteria – remains uncertain.

In this study, we aimed to assess the prevalence of US-defined abnormalities, and their association with clinical measures of disease activity, for patients with RA in clinical remission.

## Methods

### Patient recruitment

Patients with established RA were recruited as part of a study of DMARD cessation – the Biomarkers of Remission in Rheumatoid Arthritis (BioRRA) study.^5^ Patients were eligible if they were diagnosed with RA at least 12 months prior to assessment, and if they were treated with single or combination therapy using conventional synthetic DMARDs: methotrexate, sulfasalazine and/or hydroxychloroquine. Pregnant women, patients who had received glucocorticoids in the past 3 months and patients who had received any other DMARD in the past 6 months (12 months for leflunomide owing to its enterohepatic recirculation) were excluded.

For comparison, DMARD-naïve patients attending the Newcastle Early Arthritis Clinic (NEAC) undergoing clinical and ultrasonographic evaluation at first presentation with active RA (DAS28-CRP > 2.4) or seronegative non-inflammatory arthralgia (NIA) were studied as positive and negative controls respectively.^6^

### Procedures

Clinical and ultrasonographic assessment of patients was performed as previously described.^5,6^ All patients underwent clinical examination, with remission defined as a disease activity score in 28 joints (DAS28-CRP) < 2.4.^7^ Where levels of C-reactive protein (CRP) were below the detection limit of the local laboratory (<5 mg/L), a value of zero was used for the purposes of DAS28-CRP calculation. A 7-joint US scan was performed (dominant wrist, 2nd/3rd metacarpophalangeal and proximal interphalangeal joints, and 2nd/5th metacarpophalangeal joints) to quantify erosions, PD and greyscale (GS) change according to the US7 protocol of Backhaus *et al*.^8^ The US operator was blinded to the clinical disease activity scores. Erosions and tenosynovial GS (TGS) were graded as present (1) or absent (0). Synovial/tenosynovial PD (SPD/TPD) and synovial GS (SGS) were graded using a 4-point (0–3) semi-quantitative scale and summed to create total scores as previously described.^8^ All abovementioned ultrasonographic parameters were recorded for the BioRRA cohort, whereas data were not available for tenosynovial findings for the NEAC cohort.

### Statistical analysis

The association between total US scores and clinical parameters was assessed using multivariate ordinal logistic regression analyses with each individual US parameter as the dependent variable. Clinical parameters potentially correlated with US measures were selected, namely: sex, age, disease duration, smoking history, alcohol intake, rheumatoid factor and anti-citrullinated peptide antibody positivity, ACR/EULAR Boolean remission, Health Assessment Questionnaire Disability Index (HAQ-DI) score,^9^ erythrocyte sedimentation rate (ESR), and the individual components of the DAS28-CRP score. Correction for multiple testing was performed using a Benjamini–Hochberg procedure, with statistical significance defined using a significance threshold of <0.05. All statistical analysis was performed in the R statistical environment (R Core Team, version 3.3.2) using the ‘ordinal’ package.^10^

The BioRRA and NEAC studies were approved by the North East – Tyne & Wear South Research Ethics Committee (BioRRA: 14/NE/1042; NEAC: 12/NE/0251). Informed written consent was obtained from all participants in both studies.

## Results

### Patient characteristics

In total, 66, 146 and 82 patients were included within RA remission (BioRRA), active RA (NEAC) and NIA (NEAC) groups respectively (Table 1). There was no significant difference in sex or age of participants in the RA groups, although the NIA group were significantly younger with a higher proportion of females. Total SGS scores were comparable between remission and active RA, whereas total PDS score was significantly higher in the active RA group. Erosions were less commonly observed in the active RA group in keeping with the shorter disease duration. As expected, the prevalence of US-defined abnormalities was uniformly low in the NIA group.

**Table 1.** Characteristics of patients included within the analysis.

Demographic	RA remission (BioRRA) (*n* = 66)	Active RA (NEAC) (*n* = 146)		Seronegative non-inflammatory arthralgia (NEAC) (*n* = 82)	
		Value	*p*	Value	*p*
Female: *n* (%)	38 (58)	87 (60)	0.88	68 (95)	<0.01
Age: median (IQR) [range]	66 (56–71) [35–82]	59 (53–70) [17–88]	0.07	39 (32–50) [20–80]	<0.01
Years since symptom onset: median (IQR) [range]	6 (4–12) [1–40]	0 (0–0) [0–1]	<0.01	0.5 (0–1) [0–4]	<0.01
RhF positive: *n* (%)	40 (61)	87 (56)	1.00	n/a	
ACPA positive: *n* (%)	38 (58)	81 (55)	0.88	n/a	
RhF or ACPA positive: *n* (%)	47 (71)	97 (66)	0.53	n/a	
RhF and ACPA positive: *n* (%)	31 (47)	71 (49)	0.88	n/a	
Total SGS score: median (IQR) [range]	5 (3–6) [1–10]	5 (2–7) [0–14]	0.85	1 (0–2) [0–9]	<0.01
Total SPD score: median (IQR) [range]	0 (0–1) [0–7]	3 (1–5) [0–12]	<0.01	0 (0–0) [0–4]	0.07
Total TGS score: median (IQR) [range]	0 (0–1) [0–3]	nr		nr	
Total TPD score: median (IQR) [range]	0 (0–0) [0–5]	nr		nr	
Number of joints with erosion: median (IQR) [range]	1 (0–2) [0–5]	0 (0–0) [0–1]	<0.01	0 (0–0) [0–0]	<0.01
Swollen (28) joint count: median (IQR) [range]	0 (0–0) [0–2]	2 (1–6) [0–24]	<0.01	0 (0–0) [0–4]	0.75
Tender (28) joint count: median (IQR) [range]	0 (0–0) [0–2]	6 (3–10) [0–26]	<0.01	5 (3–9) [0–24]	<0.01
Patient VAS (mm): median (IQR) [range]	5 (1–13) [0–35]	51 (31–75) [0–100]	<0.01	60 (40–75) [8–100]	<0.01
CRP in mg/L: median (IQR) [range]	0 (0–0) [0–13]	12 (5–28) [0–203]	<0.01	0 (0–8) [0–156]	<0.01
ESR in mm/hr: median (IQR) [range]	9 (5–17) [1–77]*	27 (13–42) [1–122]	<0.01	9 (5–22) [2–73]	0.57
DAS28-CRP: median (IQR) [range]	1.09 (0.99–1.59) [0.96–2.34]	4.34 (3.51–5.29) [2.50–7.51]	<0.01	n/a	
ACR/EULAR Boolean remission: *n* (%)	40 (61)	0 (0)	n/a	n/a	
Total DMARDs since diagnosis: median [range]	2 [1–4]	0 [0–0]	<0.01	n/a	
Current methotrexate use: *n* (%)	55 [83%]	n/a		n/a	

*P* values are presented for comparison with RA remission group (continuous/ordinal data: Mann–Whitney *U* test; categorical data: Fisher’s exact text).

* One patient had an elevated ESR of 77 at baseline due to hypergammaglobulinaemia from secondary Sjögren’s syndrome.

ACPA, anti-citrullinated peptide antibody; ACR, American College of Rheumatology; BioRRA, Biomarkers of Remission in Rheumatoid Arthritis study; CRP, C-reactive protein; DAS28-CRP, disease activity score in 28 joints with C-reactive protein; DMARD, disease-modifying anti-rheumatic drug; ESR, erythrocyte sedimentation rate; EULAR, European League Against Rheumatism; IQR, interquartile range; n/a, not applicable; NEAC, Newcastle Early Arthritis Clinic; nr, not recorded; RhF, rheumatoid factor; SGS, synovial greyscale; SPD, synovial power Doppler; TGS, tenosynovial greyscale; TPD, tenosynovial power Doppler; VAS, visual analogue score.

### US-defined abnormalities are common in RA remission

US-defined abnormalities, particularly SGS, were common in RA remission and were equally present in patients who did or did not satisfy clinical remission regardless of whether this was defined by DAS28-CRP < 2.4 (*n* = 66) or ACR/EULAR Boolean criteria (*n* = 40) (Table 2). There were insufficient occurrences of tendon PD to permit further analysis (2/66 [3%] patients).

**Table 2.** The prevalence of US abnormalities in the RA remission group was equivalent for both DAS28-CRP and ACR/EULAR Boolean remission. Statistical significance of difference in observations between those who did and did not satisfy ACR/EULAR Boolean remission is calculated by Fisher’s exact test.

Criterion	US parameter	Remission definition		*p*
		DAS28-CRP < 2.4 (*n* = 66)	ACR/EULAR Boolean (*n* = 40)	
*n* (%) patients with total score ⩾1	SGS	66 (100)	40 (100)	n/a
	SPD	17 (26)	10 (25)	>0.99
	TGS	29 (44)	17 (43)	0.80
	Erosions	45 (68)	25 (63)	0.28
*n* (%) patients with any individual joint score ⩾2	SGS	48 (73)	27 (68)	0.27
	SPD	8 (12)	6 (15)	0.46

ACR, American College of Rheumatology; CRP, C-reactive protein; DAS28, disease activity score in 28 joints; EULAR, European League Against Rheumatism; RA, rheumatoid arthritis; SGS, synovial greyscale; SPD, synovial power Doppler; TGS, tenosynovial greyscale; US, ultrasound.

### Strong association of US and clinical parameters in active RA but not in NIA

Multivariate ordinal logistic regression was performed for each US variable within each patient group. Firstly, the association between clinical and US parameters were assessed for active RA and NIA as a measure of the face validity of the scan protocol. In active RA, swollen joint count strongly associated with both SGS [odds ratio (OR) 1.17, 95% confidence interval (CI) 1.08–1.26, adjusted *p* < 0.01] and SPD (OR 1.20, 95% CI 1.12–1.30, adjusted *p* < 0.01). In contrast, no significant associations were observed between swollen joint count and SGS or SPD in the NIA group.

### Lack of association of US and clinical parameters in RA remission

**Figure 1.**
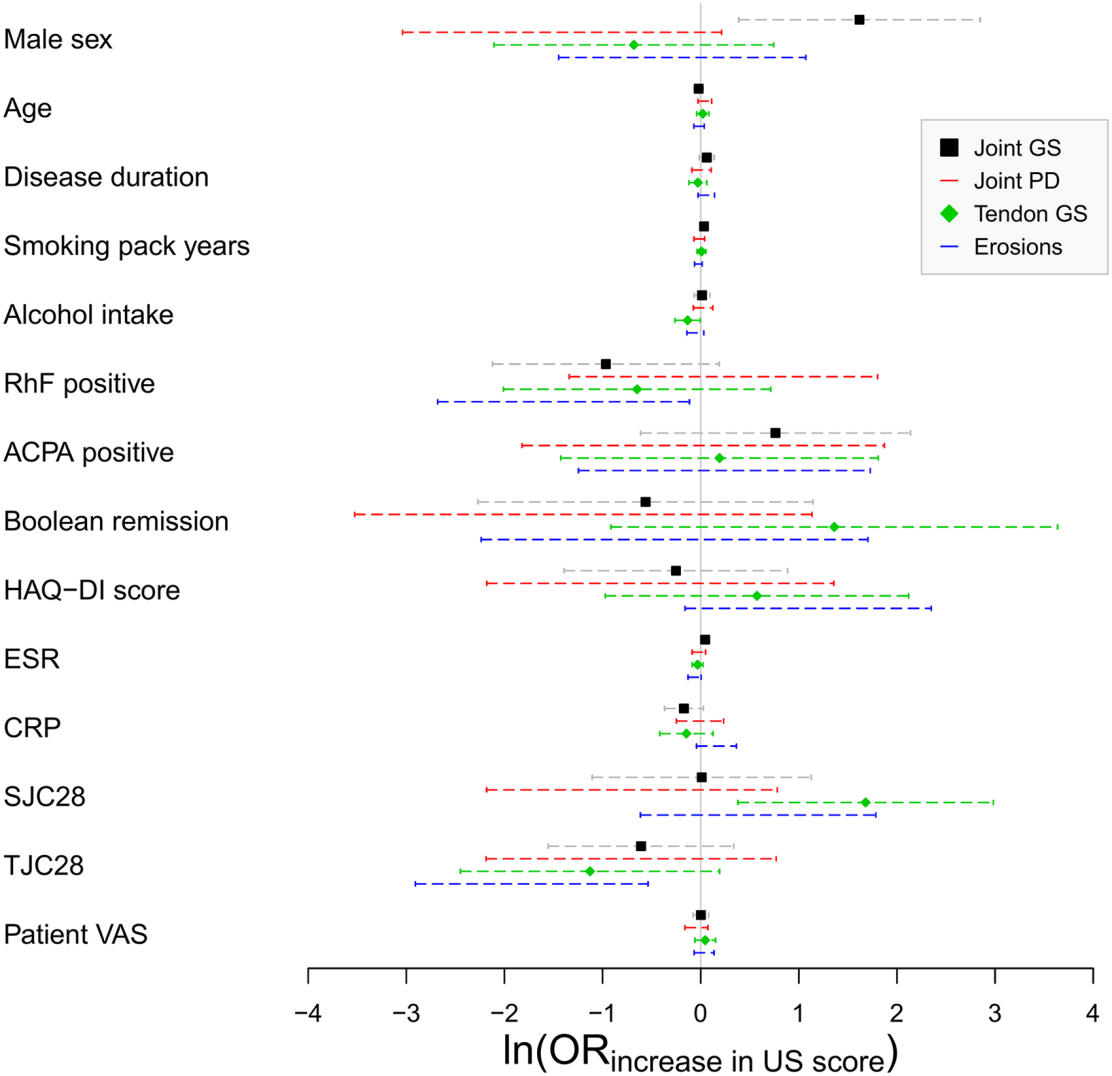
Association between clinical and US parameters in the RA remission group as assessed by multivariate logistic regression. The (ln(OR) for increase in total US score for each clinical parameter is shown, with error bars indicating the 95% CI. An ln(OR) of zero indicates no association, shown by the vertical line. Adapted with permission from Baker *et al*.^11^ ACPA, anti-citrullinated peptide antibody; CI, confidence interval; CRP, C-reactive protein; ESR, erythrocyte sedimentation rate; HAQ-DI, Health Assessment Questionnaire Disability Index; ln(OR), log-transformed odds ratio; RA, rheumatoid arthritis; RhF, rheumatoid factor; SJC28, swollen 28 joint count; TJC, tender 28 joint count; US, ultrasound; VAS, visual analogue score.

The association of clinical and US parameters was then assessed within the RA remission group (Figure 1). Six variable-score associations were statistically significant at an unadjusted *p* < 0.05 threshold: male sex and ESR *versus* total SGS score; swollen joint count and alcohol intake *versus* total TGS score; and tender joint count and rheumatoid factor positivity *versus* total erosion score (Table 3). However, none of these associations were robust to multiple test correction. Of note, no significant associations were observed between total SPD score and any of the clinical variables.

**Table 3.** The six clinical variables associated with US parameters in the RA remission group at an unadjusted *p* < 0.05 significance threshold. Corresponding adjusted *p*-values after Benjamini-Hochberg correction are also shown.

US score	Clinical parameter	OR increase in US score	95% CI	Unadjusted *p*	Adjusted *p*
SGS	Male sex	5.04	1.47–17.26	0.01	0.14
	ESR (mm/h)	1.05	1.00–1.09	0.04	0.24
TGS	SJC28	5.37	1.46–19.72	0.01	0.16
	Alcohol intake (units/week)	0.88	0.77–1.00	0.04	0.31
Erosion	TJC28	0.18	0.05–0.58	<0.01	0.06
	RhF positive	0.25	0.07–0.89	0.03	0.23

ESR, erythrocyte sedimentation rate; OR, odds ratio; RA, rheumatoid arthritis; RhF, rheumatoid factor; SGS, synovial greyscale; SJC28, swollen joint count (28 joints); SPD, synovial power Doppler; TGS, tenosynovial greyscale; TJC28, tender joint count (28 joints); US, ultrasound.

## Discussion

The past three decades have witnessed an exponential increase in the use of musculoskeletal US in the management of RA, especially as a diagnostic aid to supplement clinical examination. Strong evidence links US-defined abnormalities, in particular SPD, with active synovitis in the setting of active disease, including correlations with histological measures of inflammation in synovial tissue,^12–14^ Th17 cells in synovial fluid,^15^ and production of pro-inflammatory cytokines in *ex vivo* culture of synovial tissue.^16^ Furthermore, SPD is highly responsive to changes in disease activity,^17^ and responds quickly to treatment initiation.^18^ As such, US evaluation is broadly accepted as providing additional discriminatory value in the detection of synovitis in symptomatic individuals, especially in seronegative disease.^19^

In contrast, the role of US examination in the setting of RA remission remains uncertain, in particular the clinical significance of US-defined abnormalities in asymptomatic individuals. In this cross-sectional analysis, we demonstrate a high prevalence of musculoskeletal US abnormalities (especially SGS) in the setting of established RA remission, regardless of whether this is defined by DAS28-CRP or ACR/EULAR Boolean criteria. In a meta-analysis of 19 studies including 1369 patients in clinical remission,^20^ similarly high levels of SGS (74–86%) and combined SGS/SPD (32–44%) were observed across a range of clinical remission criteria and scan protocols. Furthermore, previous studies have shown the presence of US abnormalities at considerable levels even in healthy subjects,^21^ thus making it difficult to confidently ascribe clinically relevant thresholds for low-grade ultrasonographic findings.

Several previous studies have shown a degree of association, albeit modest, between low clinical disease activity scores and the absence of SPD;^22,23^ however, no such association has been observed in other studies.^24,25^ In our study, we first observe strong associations between swollen joint counts and SGS/SPD in active disease but not in non-inflammatory arthralgia, supporting the validity of the US7 protocol. We then demonstrate a lack of significant association between clinical and US parameters in the setting of RA remission.

Our most striking observation is the complete lack of association in RA remission between SPD and any of the clinical parameters assessed in our study, even without multiple test correction. Evidence exists to suggest that the presence of SPD, even in the setting of clinical remission, can represent ongoing subclinical synovitis. In this context, SPD has been shown to correlate with future arthritis flare,^4,26^ future bone erosions and immunohistochemical markers of synovial inflammation.^4,27^ Despite these observations, however, treatment strategies aimed at achieving ultrasonographic definitions of remission have so far failed to show superiority over standard clinical management,^28,29^ at the expense of increased adverse effects and treatment cost.^30^ Our study adds further evidence to support a lack of association between ultrasonographic and clinical measures of disease activity in the context of disease remission – a crucial disconnect which suggests a plausible explanation for the futility of US-defined treatment targets studied thus far.

There are some limitations to our study. We used a limited 7-joint scan protocol, and thus it is possible that significant US abnormalities were missed in other joints. Indeed, a strategy targeting US to symptomatic joints, and interpreting imaging within the context of the individual patient history and examination findings, may be more discriminatory for active disease though would lack the reproducibility of the validated, blinded and systematic US7 scan protocol used in this study. Corroboration of independent assessment by two ultrasonographers, as opposed to a single scan per patient, may have improved the accuracy of US assessments in this study, but was not feasible due to limited study resources. Furthermore, our study population is relatively small, and infrequent occurrences and incomplete recording of tenosynovial abnormalities restricted analysis of these variables. Differing composition of the NEAC *versus* BioRRA cohorts – namely shorter symptom duration and DMARD-naïve status in the active RA group, and younger age and increased number of females in the NIA group – may limit the generalisability of scan findings to the established remission group. Similarly, our remission group comprised exclusively patients with established disease treated with conventional synthetic DMARDs, and thus our findings may not directly translate to patients with early disease or those receiving biologic therapy. Finally, the lack of a time criterion in our remission definition prevents further analysis of ultrasonographic findings in cross-sectional *versus* sustained disease remission.

In summary, we observe substantial levels of US abnormalities in established RA clinical remission, with no significant association with clinical parameters. Most strikingly SPD, which portends a poor prognosis, failed to show association with any of the clinical parameters. Our results suggest that clinical and US examinations are non-overlapping in RA remission, challenging the proposition of US-driven management strategies in this setting.
